# Gene expression relationship between prostate cancer cells of Gleason 3, 4 and normal epithelial cells as revealed by cell type-specific transcriptomes

**DOI:** 10.1186/1471-2407-9-452

**Published:** 2009-12-18

**Authors:** Laura E Pascal, Ricardo ZN Vêncio, Laura S Page, Emily S Liebeskind, Christina P Shadle, Pamela Troisch, Bruz Marzolf, Lawrence D True, Leroy E Hood, Alvin Y Liu

**Affiliations:** 1 Department of Urology, University of Washington, Seattle, WA 98195, USA; 2 Institute for Stem Cell and Regenerative Medicine, University of Washington, Seattle, WA 98195, USA; 3 Institute for Systems Biology, Seattle, WA 98103, USA; 4 Genetics Department, University of São Paulo's Medical School at Ribeirão Preto, Brazil CEP: 14049-900; 5 Department of Pathology, University of Washington, Seattle, WA 98195, USA; 6 Current address: LEP, University of Pittsburgh Medical Center, Department of Urology, 5200 Centre Ave, Pittsburgh, PA 15232

## Abstract

**Background:**

Prostate cancer cells in primary tumors have been typed CD10^-^/CD13^-^/CD24^hi^/CD26^+^/CD38^lo^/CD44^-^/CD104^-^. This CD phenotype suggests a lineage relationship between cancer cells and luminal cells. The Gleason grade of tumors is a descriptive of tumor glandular differentiation. Higher Gleason scores are associated with treatment failure.

**Methods:**

CD26^+ ^cancer cells were isolated from Gleason 3+3 (G3) and Gleason 4+4 (G4) tumors by cell sorting, and their gene expression or transcriptome was determined by Affymetrix DNA array analysis. Dataset analysis was used to determine gene expression similarities and differences between G3 and G4 as well as to prostate cancer cell lines and histologically normal prostate luminal cells.

**Results:**

The G3 and G4 transcriptomes were compared to those of prostatic cell types of non-cancer, which included luminal, basal, stromal fibromuscular, and endothelial. A principal components analysis of the various transcriptome datasets indicated a closer relationship between luminal and G3 than luminal and G4. Dataset comparison also showed that the cancer transcriptomes differed substantially from those of prostate cancer cell lines.

**Conclusions:**

Genes differentially expressed in cancer are potential biomarkers for cancer detection, and those differentially expressed between G3 and G4 are potential biomarkers for disease stratification given that G4 cancer is associated with poor outcomes. Differentially expressed genes likely contribute to the prostate cancer phenotype and constitute the signatures of these particular cancer cell types.

## Background

The Gleason grade is a pathology characterization of prostate tumors. Tumor glands can appear from well-differentiated to poorly differentiated. The degree of differentiation is scaled from 3 to 5 (grades 1 and 2 are no longer in wide use), with 3 to indicate tumors with glandular differentiation, 4 to indicate tumors with aglandular differentiation, and 5 to indicate no differentiation with cancer cells not organized into recognizable structures. Each tumor is assigned a score of two Gleason grades. Gleason 3+3 is associated with favorable outcomes and patient survival while tumors containing 4 or 5 components have a poorer prognosis. A molecular correlate of the Gleason grade was reported where grade 3 could be distinguished from grades 4 and 5 by gene expression, whereas grades 4 and 5 were indistinguishable by gene expression [1]. Another study, however, found a different set of genes associated with degree of tumor differentiation [2]. Reis *et al*. [3] found that a higher fraction (6.6%) of intronic ncRNAs were significantly correlated to the degree of prostate tumor differentiation (Gleason score) when compared to unannotated genomic regions (1%) or from exons of known genes (2%). The difference in gene signatures reported by different laboratories has yet to be explained, but is likely due to a combination of external factors as well as actual biological difference. These studies have attempted to define a set of gene expression profiles as determined by microarray analysis that could supplement the traditional diagnostics of pathology and clinical parameters to predict disease course [4]. For example, a 5-gene model was proposed that could segregate recurrent from non-recurrent cancer [5]. Although gene signatures have been shown to associate with Gleason grade [6], none so far have been able to discriminate locally invasive from non-invasive tumors.

We have previously shown that prostate tumors contained multiple Gleason grades as well as multiple cancer cell types distinguishable by their cluster designation (CD) phenotypes [7,8]. CD antigens are cell surface molecules, and each cell type has a unique complement of these molecules. This allowed us to isolate, for example, CD26^+ ^luminal cells, CD104^+ ^basal cells, CD49a^+ ^stromal fibromuscular cells, and CD31^+ ^endothelial cells from prostate tissue samples by magnetic cell sorting (MACS) for microarray analysis using the Affymetrix GeneChip to determine cell type-specific gene expression or transcriptome [9]. These cell type transcriptomes are a powerful tool to discover lineage relationship between cell types. We applied the same cell sorting methodology here to determine the transcriptome of CD26^+ ^prostate cancer cells of Gleason 3+3 and Gleason 4+4 tumor specimens. These specimens, unlike those of 3+4 or 4+3, ensured that cells representative of only grade 3 or 4 would be obtained. The transcriptomes were then compared with each other to identify differentially expressed genes, as well as with those of the normal cell types. Furthermore, dataset comparisons between the transcriptomes of primary tumor cells and prostate cancer cell lines, which were established from metastatic lesions and showed unique CD expression [10,11], demonstrated that they were not representative of cells in primary tumors.

## Methods

### Prostate tumor tissue and cell sorting

Anonymized tissue samples were obtained at radical prostatectomy under approval by the University of Washington Institutional Review Board. Pertinent pathology information was requested from a research coordinator who was authorized to access the patient database in the Department of Urology. The resected glands were cut into 3-mm thick transverse blocks, and frozen sections from representative right and left areas of the apex, middle and base regions were stained to locate the tumor foci. With the stained sections as guide, tumor tissue samples were excised from the corresponding regions and placed in sterile tubes. Processing of tissue pieces for cell sorting was as described [9,12]. The collected samples were minced and digested by collagenase type I (Invitrogen, Carlsbad, CA) in serum-free RPMI1640 media supplemented with 10^-8 ^M dihydrotestosterone on a magnetic stirrer at room temperature overnight. The resultant cell suspension was filtered with 70-μm Falcon cell strainer to remove debris, diluted with equal volume of Hanks balanced salt solution (HBSS), and aspirated with 18-gauge needle. Cells were pelleted by centrifugation and the digestion media was analyzed by Western blotting for TIMP1 protein [13] to gauge purity of the cancer specimens. The single cell preparation was partitioned into stromal and epithelial fractions on discontinuous Percoll density gradients (Amersham Pharmacia, Piscataway, NJ). Cells banding at the epithelial density were collected and used for sorting with CD26 antibody. The tumor specimens of 05-179 and 08-032 weighed 0.6 g and 6 g respectively, and were above the minimal weight requirement for cell sorting. The pathology characteristics of tumors from which cancer cells were obtained were as follows - 05-179: Gleason 3+3, T2cN0Mx, 4.7 cc tumor volume; and, 08-032: Gleason 4+4, T3b, 27 cc tumor volume.

### MACS cell isolation and FACS analysis of sorted cells

Gradient-purified epithelial cells were labeled with phycoerythrin (PE)-conjugated anti-CD26 (1:20; M-A261, BD PharMingen, San Diego, CA) and sorted by MACS (Miltenyi Biotec, Auburn, CA) as described previously for the normal cell types [9]. Aliquots of positive and negative cell fractions were analyzed by fluorescence activated cell sorting (FACS, Becton Dickinson, Mountain View, CA) to monitor sort efficiency as reported previously; only >85% CD26^+ ^fractions were used for microarray analysis [14]. The sorted cancer cells were pelleted by centrifugation and total RNA was extracted using RNaqueous (Ambion, Austin, TX).

### Prostate cancer cell lines

Prostate cancer cell lines LNCaP, C4-2, CL1 (and its subclones CL1.1 and CL1.31), DU145 and PC3 were cultured and harvested for array analysis as described in our previous report on differential gene expression between LNCaP and C4-2 [15].

### Immunohistochemistry

Serial 5-μm sections were prepared from frozen blocks, fixed in cold acetone, and processed for immunohistochemistry using a three-step indirect avidin-biotin-peroxidase procedure. The primary antibodies used were the CD antibodies described in our previous reports [7,16]. Anti-Dlx1 (clone AB5724, Chemicon/Millipore, Billerica, MA), was used at 1:500. Sections were counterstained with hematoxylin. The immunostained sections were imaged with Olympus BX41 microscope (Olympus, Melville, NY) equipped with MircoFire digital camera (Optronics, Goleta, CA). Images were processed with Photoshop CS (Adobe Systems, San Jose, CA).

### Western blot analysis

Cell-free tissue digestion media preparations (g tissue/ml media) diluted in equal volume of HBSS were resolved by gradient polyacrylamide gel electrophoresis and transferred to membrane filter for probing by AGR2 antibody (1:500; 1C3, Abnova, Taipei, Taiwan). The 17-kDa AGR2 protein was visualized using ECL (GE Healthcare, Piscataway, NJ). Prostate specific antigen (PSA) antibody (1:1,000; A67-B/E3, Santa Cruz Biotechnology, Santa Cruz, CA) was used as sample loading control.

### DNA array analysis

Quality and concentration of RNA were determined using Agilent 2100 Bioanalyzer and RNA Nano or Pico Labchip (Agilent Technologies, Santa Clara, CA) where appropriate. Only RNA samples of sufficient concentration and showed no degradation as evidenced by distinct 18S and 28S ribosomal bands were used for microarray experiments. The Human Genome U133 Plus 2.0 GeneChips (Affymetrix, Santa Clara, CA) were used for expression profiling. The U133 array contains probesets representing 54,675 genes, splice variants, and ESTs. The GeneChips were prepared, hybridized, and scanned according to the protocols provided by Affymetrix (P/N 702232 Rev. 2) [9,15]. In brief, 200 ng RNA was reverse transcribed with poly (dT)/T7 promoter primer, and the cDNA was made double-stranded. *In vitro *transcription was performed with biotinylated ribonucleotides, and the biotin-labeled cRNA was hybridized to the GeneChips. The chips were washed and stained with streptavidin-PE using FS-450 fluidics station (Affymetrix). Data was collected with Affymetrix GeneChip Scanner 3000. A total of three CD26^+ ^prostate cancer array datasets were obtained from two patients; with 05-179 analyzed twice and 08-032 analyzed once. Two replicates were run for each prostate cancer cell line: CL1, CL1.1, CL1.31, DU145 and PC3. Datasets previously obtained from five non-sorted whole-tissue tumor specimens and their matched non-cancer specimens were used for comparison [14]. All transcriptome datasets are deposited in our public UESC database http://scgap.systemsbiology.net/, which makes them available for data query [17].

### Computational analysis of datasets

For differential gene expression, datasets were analyzed by HTself, a self-self based statistical method for low replication microarrays [18] using a strategy very similar to that described by Pascal *et al*. [14]. To apply this method, all possible combination of pair-wise comparisons among experiments were taken to create sets of ratios. Gene expression level was defined as the normalized and summarized intensities of each GeneChip probeset, and was presented as its logarithmic value: *X *= log_2_(Normalized intensity). This step was carried out using the standard robust multi-array average (RMA) method [19], implemented in the analysis pipeline SBEAMS [20]. The strength of differential expression between any pair of experiments was estimated by *M*_*i *_= log_2_(ratio) = *X_i_*-*X_a_*, where *a *represented one particular cell type and *i *represented each given cell type in the set: B (basal), L (luminal), S (stromal), E (endothelial), G3 (Gleason 3) or G4 (Gleason 4). To estimate reliability, the HTself method used virtual self-self experiments (i.e., G3 cancer *vs*. G3 cancer) to derive intensity-dependent cutoffs. Accordingly, a probeset was considered significantly differentially expressed if at least 80% of its log-ratio combinations were outside the 99.9% credibility intensity-dependent cutoff. Moreover, an average greater than 8-fold difference in expression level was chosen. The computational analysis results were verified by dataset query of known differentially expressed genes. In principal components analysis (PCA) of the various transcriptome datasets, a gene expression subspace was obtained that brought out the principal sources of variability among transcriptomes of the different prostate cell types. The rotation matrix was obtained by using averages of *X_G3_*, *X_G4_*, *X_L_*, *X_B_*, *X_S_*, and *X_E_*, and these were plotted as projections on the principal components space. The various transcriptomes were then projected into this PCA-generated subspace, which could be rotated freely to visualize the spatial separation of the individual nodes. Pathway analysis of selected genes was done with KEGG [21].

## Results

### Gene expression of cancer cells of a Gleason 3+3 tumor

**Figure 1:**
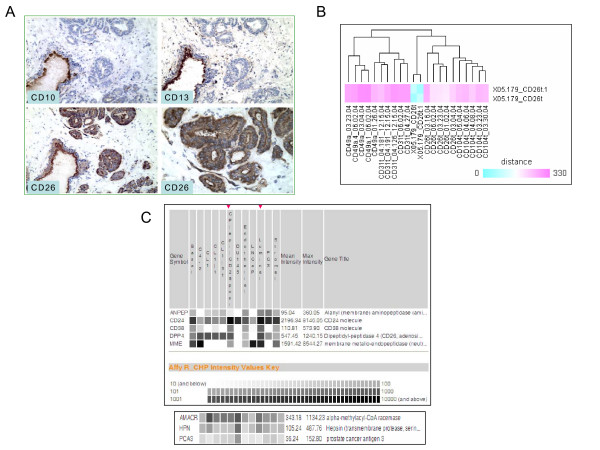
**Prostate cancer cell gene expression**. **A: **Cancer CD phenotype. Serial sections of human prostate from specimen 05-179 were stained for CD10, CD13, and CD26. Cancer glands in these serial sections are not stained for CD10 (top left) and CD13 (top right), whereas a single benign gland (lower left in the photomicrographs) is; original magnification 200×. Cells in the cancer glands, like luminal cells of benign glands, are positive for CD26 (bottom left). In the higher magnification (400×, bottom right), the cancer cells show the characteristic prominent nucleoli. **B: **Dataset clustering. The relationship of the CD26^+ ^cancer cell transcriptome to that of CD49a stromal, CD31 endothelial, CD26 luminal or CD104 basal is shown. Whole transcriptomes were used to generate Euclidean distances. The self replicates of CD26^+ ^cancer (blue) are in the middle. **C: **Gene expression in virtual Northern blot format. Affymetrix signal values are represented on a gray scale. The cell type-specific transcriptomes are listed on the top with red arrowheads to identify CD26^+ ^cancer and luminal. C4-2, CL1, CL1.1, CL1.31, DU145, LNCaP, PC3 are cancer cell lines.

CD26^+ ^prostate cancer cells were isolated from tumor specimen 05-179, split into two aliquots, and each was analyzed by DNA array. The 05-179 tumor was scored Gleason 3+3 with negative margins (i.e., with no evidence of extraprostatic spread). Cancer cells of the 05-179 tumor glands were characterized as CD26^+^/CD13^-^/CD10^- ^(Figure 1A), the predominant cancer cell type with a frequency of ~70% [7]. Absent expression of CD10 and CD13 distinguished these cancer cells from CD10^+^/CD13^+ ^luminal cells. Prostate basal cells were negative for CD10, CD13 and CD26, and basal cell CD markers were not detected in the cancer by immunostaining (data not shown). Immunostaining of all representative 05-179 tissue sections containing cancer did not detect the CD10^+ ^cancer cell type, which has a frequency of ~30% [7].

For the single specimen of 05-179 cancer, statistical analysis of gene expression with traditional methods was not applicable. Instead, HTself was used to analyze for differential gene expression between the CD26^+ ^G3 cancer transcriptome and the previously determined transcriptome of the normal counterpart, CD26^+ ^luminal [9]. Cluster analysis comparing the transcriptome datasets of CD26^+ ^G3 cancer cells and the previously determined principal cell types of the prostate [9] was summarized (Figure 1B). The gene expression profile of CD26^+ ^cancer was closer to that of luminal than basal, in agreement with previous CD phenotype analysis [7] and cell type-specific expression data of cytokeratin subtypes [22].

The array data was reported as RMA-normalized Affymetrix signal intensities implemented in SBEAMS or as a composite value *M*_*i *_= log_2_(ratio) = *X*_*i*_-*X*_*a*_, where *a *represented cancer and *i *represented normal. For dataset query, we enabled simple searches to display results in downloadable formats as previously described [17]. Query results of signal intensity values were graphically shown on a grayscale. Gene expression levels determined by microarray for CD10/MME, CD13/ANPEP, CD24, CD26/DPP4, and CD38 were concordant with immunohistochemistry, i.e., CD26^+^/CD13^-^/CD10^-^; increased CD24 and decreased CD38 compared to luminal cells (Figure 1C). The CD26^+ ^cancer cells also had elevated expression of prostate cancer-associated genes AMACR (α-methylacyl-CoA racemase), HPN (hepsin) and PCA3 (prostate cancer antigen 3). These results indicated minimal contamination of CD26^+ ^luminal cells from non-cancer tissue that might be present in the tumor specimen. In the same display, the expression profiles of these CD in the various prostate cancer cell lines were in agreement with their CD expression previously determined by flow cytometry [10,11]. For example, LNCaP and C4-2 are CD10^+^; C4-2 differs from LNCaP in being CD26^+^; PC3 is CD13^+^, DU145 is CD24^+^. Overall, the gene expression and CD phenotype of the CD26^+ ^cancer cells differed substantially from those of the cancer cell lines indicating that it is unlikely that any of the cell lines, which were established from metastatic lesions, could have derived from the CD26^+^/CD10^-^/CD13^- ^cancer cell type found in a majority of tumors.

Differential gene expression between CD26^+ ^G3 cancer cells and CD26^+ ^luminal cells analyzed by HTself identified 121 genes with increased cancer expression and 86 genes with decreased cancer expression by at least 8-fold relative to luminal cells (see Additional File 1 for full list, and Additional File 2 for self-self experiment and HTself cutoff for differential expression). Although they were up-regulated, AMACR and HPN were not among those with the highest fold-increase in expression in this set. BRE, which encodes a death receptor-associated anti-apoptotic protein with an inhibitory function in the mitochondrial pathway, was the most up-regulated, followed by FLJ242562, R55784, MMP12, and KCNH8. IL24, which was highly up-regulated in this dataset, was recently found to be up-regulated in tumor-associated stromal cells as well [14]. The many ESTs (highlighted in tan, Additional File 1), e.g., KIAA0960, also contributed to the signature of this prostate cancer cell type. Several Wnt pathway genes (highlighted in yellow, Additional File 1) were identified. Down-regulated genes by at least 8-fold relative to luminal cells included peptidases [ENPEP (glutamyl aminopeptidase), CPM (carboxypeptidase M), in addition to ANPEP/CD13 and MME/CD10], TNFRSF19 (involved in apoptosis), ADD3 (adducin, important for cell-cell contact in epithelial tissues), GEDP (the gene differentially expressed in the prostate), and MSMB/PSP_94_. MSMB, KLK3/PSA and ACPP (prostatic acid phosphatase) constitute the well-known three most abundant prostatic secretory products. Unlike KLK3 and ACPP, luminal expression of MSMB appears not to be regulated by stromal influence [12], and is known to be down-regulated in cancer cells [23].

### Gene expression of cancer cells of a Gleason 4+4 tumor

The 08-032 tumor was scored Gleason 4+4, margin positive with seminal vesicle involvement. The Gleason scoring of 08-032 indicated that virtually all the cancer cells represented the Gleason grade 4 (G4) type while those of 05-179 the G3 type (no frozen tumor tissue block was available for 08-032 for CD immunohistochemistry). Verification of the "purity" of this rather large (6 g) tumor specimen, i.e., absence of non-cancer, was determined by Western blot analysis of tissue collagenase digestion media for TIMP1 protein (data published in ref. [14]). TIMP1 protein is produced by luminal cells and not cancer cells [13]. Specimen 08-032 showed minimal reactivity for TIMP1, indicating a relatively pure sample with little contamination by non-cancer. Differential gene expression was analyzed between G3 and luminal cells, and between G4 and G3 cells.

**Figure 2:**
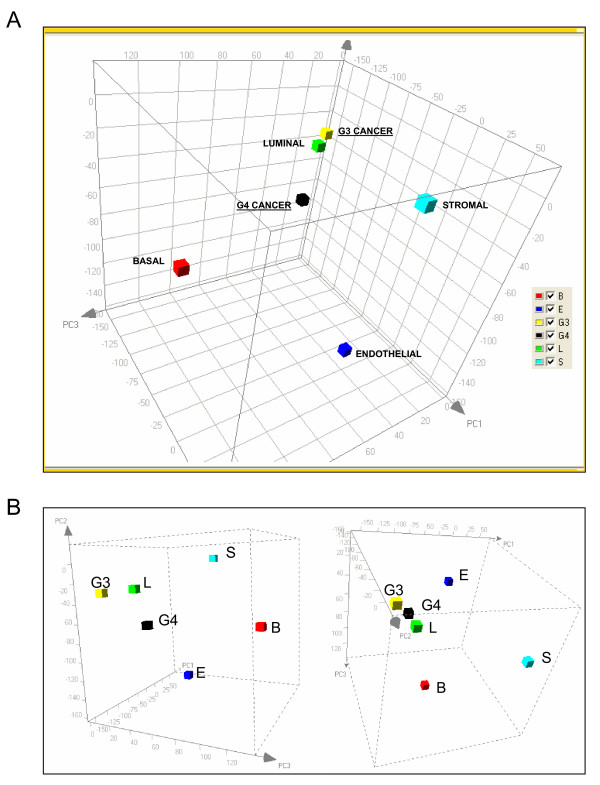
**PCA projections of Gleason 3 and Gleason 4 cancer transcriptomes**. **A: **Three-dimensional projection of G3 and G4 transcriptomes with respect to those of prostate cell transcriptomes for stromal cells (S), luminal cells (L), endothelial cells (E), and basal cells (B) in a PCA-derived subspace. **B: **Two other orientations from a different point of perspective of the PCA space. The 3D coordinate system was obtained by performing the usual PCA, defining the rotation matrix related to the top three principal components and applying it to all datasets to create a subspace that highlights the expression particularities of each prostate cell type.

PCA (Figure 2) displayed the relatedness of the G3 and G4 transcriptomes to each other and to those of the other previously determined major cell types of the prostate: stromal (S), luminal (L), basal (B), and endothelial (E). Their particular placement suggested that, with respect to the major differences in gene expression of cell types, G3 and G4 were most similar to luminal (L) than any of the other histologically normal prostate cell types. G4 was less similar to luminal (L) than G3 as indicated by the longer separation between the respective data points. Neither the G3 nor the G4 transcriptome clustered near that of basal cells (B) indicating that these CD26^+ ^G3 and G4 cancer cells were more characteristic of luminal cells than basal cells in gene expression as well as CD phenotype.

### Comparison of cancer cell and cancer tissue transcriptomes

**Figure 3:**
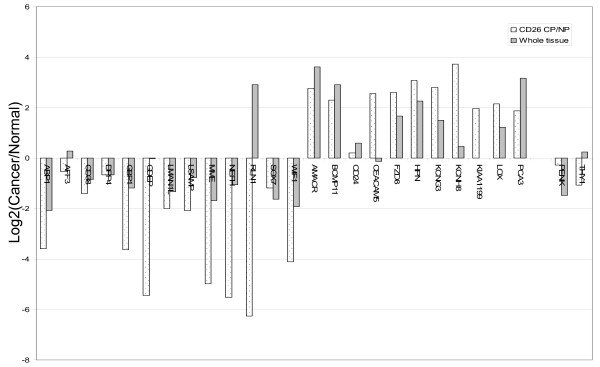
**Cancer cell *vs*. cancer tissue transcriptomes. **Profile of genes with ≥ 8-fold difference in expression in cancer compared to normal luminal cells as determined by analysis of sorted CD26^+ ^(NP and CP) cells and whole tissue transcriptomes (NP and CP). Positive log2(Cancer/Normal) on the *y*-axis indicates increased cancer expression while negative log2 indicates decreased cancer expression.

The combined CD26^+ ^cancer cell transcriptome datasets were compared to those of five matched cancer (CP) and non-cancer (NP) tissue specimens obtained also via Affymetrix GeneChips by us [14]. Pathology characteristics of the CP tissue specimens are listed in Table 1. Cell sorting was not possible for these CP samples because of insufficient specimen quantity received (<0.5 g) for MACS. Additionally, the presence of significant non-cancer in the samples (as indicated by TIMP1 Western) or that of multiple CD cancer cell types (e.g., CD10^+^), especially in the higher Gleason grades [7,8], was also problematic. Some of the most differentially expressed genes identified by HTself in cancer cells were compared to gene expression levels in whole tissue specimens (Figure 3). Data was reported as a composite value of RMA-normalized Affymetrix signal intensities: *M_i _*= log_2_(ratio) = *X_i_*-*X_a_*. For most, there was concordance between sorted cell and tissue expression (e.g., down-regulation of ABP1 and up-regulation of BCMP11 in cancer). The main apparent discordance was seen in genes analyzed as down-regulated by cell datasets (AFF3, GDEP, RLN1) but not by tissue datasets. This could be explained by the likelihood that these genes were expressed by cell types other than luminal or cancer epithelial in the tissue specimens. Expression of stromal cell genes PENK [24] and THY1/CD90 [14] was minimal indicating that CD26 sorting was efficient enough to exclude CD90^+ ^stromal cells [14], the other major cell type of tumor tissue. These results also indicated that processing for MACS did not significantly affect gene expression.

**Table 1:** Prostate tumors used to generate CP tissue expression datasets.

case	Gleason	stage	tumor volume (cc)
05-206	3+4	T3a	6
05-213	3+4	T2c	3.4
05-215	3+4	T2c	2.5
05-218	4+5	T3a	3
05-220	4+5	T3aN1	>5

### Wnt signaling in prostate cancer

FZD8, WIF1 and SOX7 were identified by HTself as differentially expressed between cancer and luminal cells, and in whole tissue CP compared to NP (see Figure 3 and Additional File 1). In the cancer cells as well as cancer cell lines (data not shown), there was increased expression of Wnt receptor FZD8; decreased expression of Wnt inhibitor WIF1, which functions to sequester Wnt molecules from receptor binding, and that of the transcription factor SOX7. Stromal expression of WNT2 and SFRP4 suggests Wnt signaling between stromal and epithelial cells in the prostate. SFRP4 was also recently found to up-regulated in tumor-associated stromal cells [14]. FZD8, WIF1, SOX7 identified in our analysis appeared to contribute to an elevated expression of genes controlled by the Wnt molecule (increase in receptors and decrease in inhibitors). Uncontrolled Wnt signaling had been reported in many types of cancer, including prostate [25]. Here, we identified three key members of this signaling pathway that were abnormally expressed in the CD26^+ ^cancer cells.

### Transcription factors with increased expression in cancer

**Figure 4:**
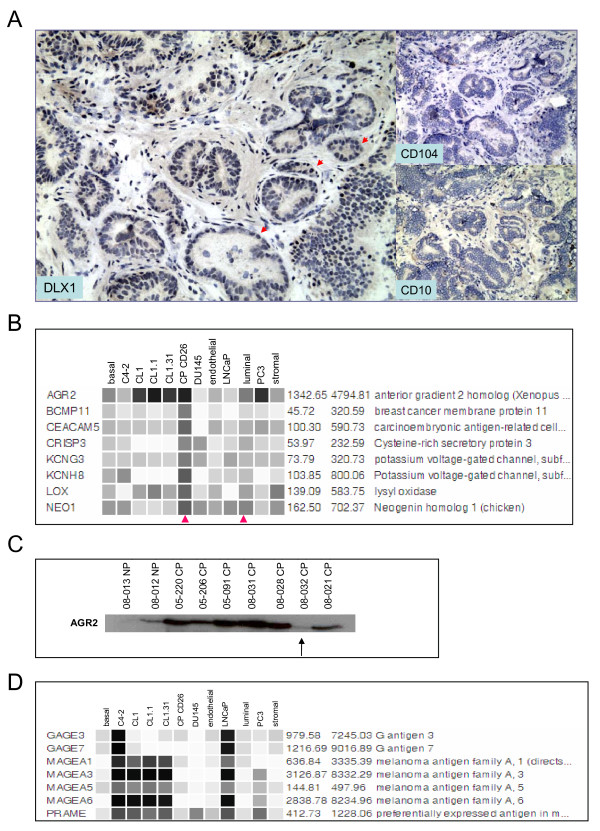
**Prostate cancer genes**. **A: **Cancer expression of DLX1. Immunohistochemistry shows positive nuclei (three examples indicated by red arrows, left panel) of cancer cells (case 04-030E) strongly stained. Cancer glands are negative for basal CD104 (top right) and luminal CD10 (bottom right); original magnification 200×. **B: **Candidate prostate cancer biomarkers. Genes with increased expression (based on array signal values as used in Fig. 1C) in CD26^+ ^cancer *vs*. luminal (arrowheads) that encode secreted - AGR2, CEACAM5, CRISP3, NEO1, membrane anchored - LOX, BCMP11 or transmembrane - KCNH8, KCNG3 proteins are shown. **C: **Western blot verification of AGR2 expression. In nearly all cases of CP except 08-032 (indicated by arrow), AGR2 protein was detected at higher levels. AGR2 in any matched NP samples may be derived from diffusion from the cancer foci. **D: **Candidate biomarkers from LNCaP. Through dataset analysis, these genes were found differentially expressed between CD10^-^/CD26^+ ^cancer and CD10^+^/CD26^- ^LNCaP.

The up-regulation and down-regulation of genes in the cancer cells could also be attributed to the activation of specific transcription factors such as ATF3, RUNX2, ERG, DLX1 (and DLX2) identified in the cancer cell transcriptomes. The fold increase in CD26^+ ^cancer cells over luminal cells for ATF3 = 9, RUNX2 = 9, ERG = 21, DLX1 = 11 was similar to that for AMACR = 12 and HPN = 8. Prostate cancer up-regulation has been previously documented for ATF3 [26], RUNX2 [27] and ERG [28]. ATF3 is involved in cell proliferation and survival, and is regulated by androgen in prostate cancer cells. RUNX2 is overexpressed in cancers that, like prostate cancer, metastasize to bone. ERG is the fusion partner of TMPRSS2 in prostate cancer [29]. DLX1 may be involved in epithelial-neuronal cell conversion [30], as prostate cancer not infrequently displays neuroendocrine differentiation. Immunohistochemistry verification of DLX1 gene expression levels with anti-DLX1 showed protein expression was localized to the cancer cell nuclei (Figure 4A).

### Prostate cancer biomarkers

Genes encoding secreted or extracellular proteins with increased expression in prostate cancer may serve as potential biomarkers for diagnosis or prognosis. Several genes that encode these proteins were upregulated in the CD26^+ ^cancer cells: AGR2, BCMP11, CEACAM5/CD66e, CRISP3, KCNG3, KCNH8, LOX and NEO1 (Figure 4B). These proteins could be measured in biospecimens by using, for example, quantitative proteomics methods. Except for AGR2 (anterior gradient 2, signal intensity at 4168 *vs*. 77 in luminal), their signal intensity indicated that their abundance was in the moderate class (e.g., 268 for BCMP11; 217 for CRISP3). BCMP11 (breast cancer membrane protein, also known as AGR3) was originally discovered by proteomics in breast cancer cell lines T-47D and MCF-7 [31]. BCMP11 was specific to cancer and not found in normal ductal epithelial cells. The related AGR2 was previously shown to be androgen-inducible and overexpressed in prostate cancer [32,33]. A ~19-kDa AGR2 protein was detected in cancer tissue preparation by mass spectrometry of 2-D gel electrophoresis spot [34]. Western blot analysis of CP *vs*. NP media confirmed overexpression of AGR2 protein in cancer (Figure 4C). Note that the lower level of AGR2 protein in 08-032 CP was concordant with decreased expression of AGR2 in this G4 cancer transcriptome (see below). LOX and NEO1 also appeared to be expressed by NP stromal cells, which would make them less suitable as cancer biomarkers.

### Genes potentially associated with node metastasis

The cancer cell transcriptomes were compared to the cancer cell lines for markers potentially associated with advanced disease. These cells have been in culture for many years and have undergone changes as a result; however, the expression of certain genes related to prostate cancer metastasis could still be maintained. For example, for lymph node metastasis, potentially important genes could be identified through a comparison of the CD26^+ ^cancer and LNCaP transcriptomes. Node metastases are CD10^+^, whereas the CD26^+ ^cancer cells are CD10^-^, so it is possible that LNCaP is a representative of the CD10^+ ^cancer cell type found in ~30% of prostate tumors and nearly all positive nodes [8]. The two datasets were analyzed to identify genes differentially expressed by ≥8-fold. All genes known to be involved in CELL PROLIFERATION as annotated in GeneOntology were filtered out. There are other genes whose expression appeared to be linked to cell culturing [35], but because they belong to different functional categories they were not filtered out. Genes with increased expression in LNCaP (and its lineage-related C4-2, which has extensive shared gene expression [15]) were examined in CD26^+ ^cancer. Figure 4D shows the expression of cancer/testis melanoma antigens MAGE and GAGE. Expression of GAGE-7 in LNCaP was reported in the literature [36]. Kufer *et al*. [37] reported that MAGE expression in circulating epithelial cells was detected significantly more in patients carrying a higher risk of disease recurrence than those with a lower risk. Patients with metastasis had higher MAGE expression compared to those with localized disease. There was also concordance in expression between primary tumors and bone marrow. In line with these genes, a gene called preferentially expressed antigen in melanoma (PRAME) was also scored in LNCaP and C4-2. Other candidates included DDC, MANEA, ZWINT, while ERG and SERPINA3 were examples of genes scored with decreased expression in LNCaP compared to CD26^+ ^cancer. Genes with the least fold difference in expression, not unexpectedly, were those encoding ribosomal proteins. For pathway visualization, the LNCaP>CD26^+ ^cancer analysis result was uploaded to KEGG. Two notable networks for the data were ANDROGEN AND ESTROGEN METABOLISM and FATTY ACID METABOLISM. It is known that androgens and lipid metabolism are coupled in LNCaP cells leading to accumulation of neutral lipids [38].

### Comparison between Gleason 3 and Gleason 4 cancer cells

**Figure 5:**
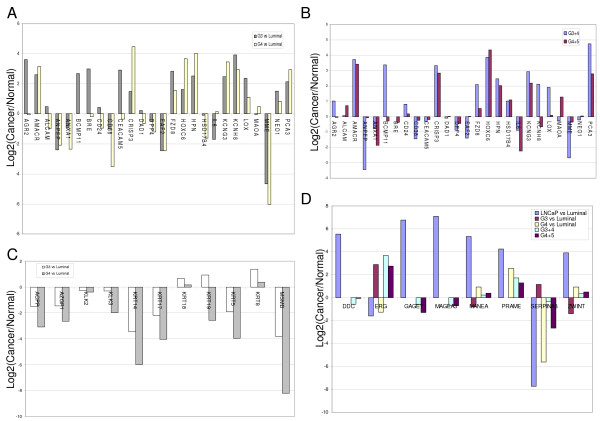
**Differential gene expression between Gleason 3 and Gleason 4 transcriptomes**. **A: **Expression levels of selected genes in G3 and G4 relative to luminal cells. **B: **Expression levels of these G3 and G4 genes in G3+4 *vs*. G4+5 tumors. **C: **Differential expression of epithelial genes in G3 and G4 relative to luminal cells. **D: **Display for LNCaP genes in primary tumors. Positive log2(Cancer/Normal) on the *y*-axis indicates increased cancer expression and negative log2 indicates decreased cancer expression.

HTself analysis of differentially expressed genes between G3 and G4 identified 147 genes with increased expression and 197 genes with decreased expression by at least 8-fold in G3 relative to G4 cells (see Additional File 3 for full list). In this set, the genes with the highest fold differential expression included many of the secreted biomarker candidates (*cf*. Figure 4B). Their expression ratios in G3 *vs*. G4 cells were: AGR2 = 13.4, BCMP11 = 18.6, CEACAM5 = 8.6, CRISP3 = 0.1, KCNG3 = 0.5, KCNH8 = 2.0, LOX = 2.5, NEO1 = 1.6 (Figure 5A). For most secreted biomarker candidates, expression was lower in G4 cells. The relative expression of ANXA1 in the CD26^+ ^cancer cells *vs*. luminal cells was also decreased in G4 compared to G3, whereas relative IL6 expression was increased. Decreased expression of ANXA1 has been associated with prostate cancer and was postulated to enhance tumor aggressiveness via the induction of IL-6 [39]. Loss of the androgen-regulated tumor suppressor EAF2, which has been reported in high grade tumors [40], was also evident in both G3 and G4 cells. Increased CRISP3 immunostaining in grade 4 (8.5-fold) was reported by Bjartell *et al*. [41]. Note also the decrease in BRE and the loss of CD38 expression, which has as one of its functions to make a cell susceptible to apoptotic signals. Differential CD38 staining has been reported in prostate cancer by us [7]. Like CRISP3 and KCNG3, AMACR, HOXC6, HPN, and PCA3 expression was increased in G4 compared to G3. The G3/G4 ratios were: AMACR = 0.7, HPN = 0.3, PCA3 = 0.6; plus DAD1 = 1.8, HSD17B4 = 3.1, MAOA = 0.7, three of the markers reported to be up-regulated in grade 4 and 5 [1]. Of the 5-gene signature presented by Singh *et al*. [6], only HOXC6 showed a 5-fold increase in our G4 dataset. Although the whole tissue transcriptomes were derived from tumors of mixed cancer cell types, they could be grouped into two Gleason categories: three of Gleason 3+4, and two of Gleason 4+5 tumors. Relative differential expression with respect to higher Gleason score was verified for the down-regulation of biomarker candidates AGR2, ANXA1, BCMP11, BRE, CD24, FZD8, KCNH8, LOX and the up-regulation of ANPEP, MME, HOX6C, and MAOA (Figure 5B).

The expression of epithelial genes such as ACPP, AZGP1 (zinc α2-glycoprotein), KLK2/hK2, KLK3, and MSMB showed a trend toward down-regulation relative to luminal cells (Figure 5C). With regard to the epithelial cytokeratins (KRT), relative expression levels of luminal cell KRT8 and KRT18 were increased in G3 and G4, whereas those of basal and intermediate cell KRT5, KRT14, KRT17 were significantly decreased. KRT19, which is expressed by both basal and luminal epithelial cells and is thought to be a marker of the transit-amplifying population [42], was slightly increased in G3, but decreased in G4 with respect to luminal cells.

The LNCaP genes as potential markers of metastatic potential were also queried between G3 and G4 (Figure 5D). Note the decrease in ERG (2.3-fold) and SERPINA3 (53.5-fold) in G4 cells as that found in the G3/LNCaP comparison. Of the others, PRAME showed a 13.6-fold increase in G4. No expression of MAGE or GAGE was seen.

For pathway visualization, the G3 *vs*. G4 cancer analysis result was uploaded to KEGG. Notable networks for the data included EXTRACELLULAR MATRIX-RECEPTOR INTERACTION and CYTOKINE-CYTOKINE RECEPTOR INTERACTION. One of the most differentially up-regulated genes in G4 cells was CCL3 (chemokine C-C motif ligand, Additional File 3). CCL3 encodes the α subunit of MIP1 (macrophage inflammatory protein-1), which is active in recruitment and activation of polymorphonuclear leukocytes in acute inflammation. The β subunit gene, CCL4, was also differentially expressed in G4. CCL4 was recently reported to be associated with prostate cancer recurrence by Blum *et al*. [43].

### Comparison with other cancer datasets

**Figure 6:**
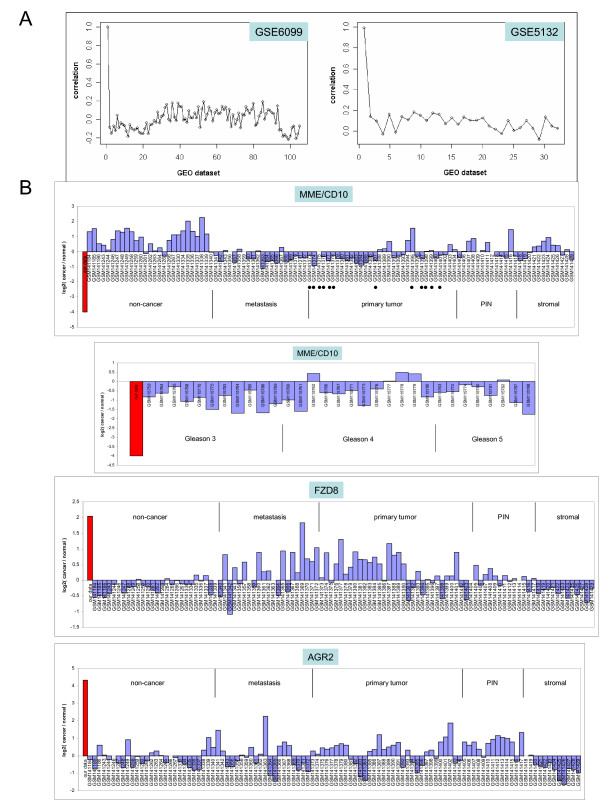
**Dataset comparison to 05-179 Gleason 3 transcriptome**. **A: **Dataset correlation. Each node represents a single dataset with the first data point being the 05-179 CD26^+ ^cancer transcriptome (in red). In the GSE6099 display, the flanking lowest correlates are those of non-cancer and stromal cells (see B). **B: **Single-gene query displays. Shown are those for MME/CD10 in both GSE6099 (top), where the laser-captured Gleason grade 3's are indicted by black dots, and GSE 5132 (bottom); FZD8 and AGR2 in GSE6099. Positive log2(Cancer/Normal) on the *y*-axis indicates increased cancer expression and negative log2 indicates decreased cancer expression.

Two publicly available high throughput gene expression datasets available for comparison with our data were GSE6099 [44] and GSE5132 [1]. The GSE6099 dataset is comprised of n = 104 laser-capture microdissected (LCM) cells for non-cancer, premalignant prostatic intraepithelial neoplasia (PIN), primary tumor, and metastasis analyzed against non-cancer prostate RNA obtained from a commercial source in two-color cDNA arrays. The arrays contained 20,000 probes mapped to 9,704 known genes, from which the overlap with the HU133 array was 7,698 genes. The GSE5132 dataset is comprised of n = 30 LCM cells for non-cancer, Gleason grades 3, 4 or 5 cancer analyzed against normal adjacent glands in two-color cDNA arrays. The arrays contained 15,488 probes mapped to 3,615 known genes, from which the overlap with HU133 was 3,135 genes. The overall correlation (~0.2) between the CD26^+ ^G3 cancer dataset and these, which included Gleason 3 cancer, was rather poor (Figure 6A). It is important to bear in mind that the comparison involved different array platforms (Affymetrix DNA *vs*. cDNA arrays), cell isolation methods (MACS *vs*. LCM), cancer/normal comparison (sorted normal cells *vs*. commercial RNA of normal *vs*. adjacent normal glands). When GSE6099 and GSE5132 were compared for the best correlation possible, the result was also poor with a mean of 0.25 (range: 0.16-0.36 for all possible permutations). We created an Excel-based query tool to display the expression pattern of a particular gene across all these datasets (Figure 6B). For example, MME/CD10 was down-regulated in the CD26^+ ^G3 cancer cells, and both GSE datasets showed agreement (some expression was detected in the stromal cells of GSE6099). The fold decrease was largest between CD26^+ ^G3 cancer cells and luminal cells, as possible from a cell-to-cell comparison. FZD8 was shown up-regulated in CD26^+ ^G3 cancer cells, and the GSE6099 dataset also showed agreement; GSE5132 contained no information regarding FZD8. For the candidate secreted biomarkers, there was no information for CRISP3, KCNG3 or KCNH8 in GSE6099; for BCMP11, CEACAM5, CRISP3, KCNG3 or LOX in GSE5132. AGR2 in the GSE6099 dataset is shown, and increased expression was detected in PIN. Immunohistochemistry had previously shown AGR2 expression in high-grade PIN [32].

## Discussion

Transcriptome analysis of CD26^+ ^prostate cancer cells demonstrated that it was possible to isolate and characterize gene expression in cancer cells from solid tumors weighing 0.5 g or more utilizing our cell sorting methodology. The extensive tissue processing also did not appear to alter substantially the gene expression profiles of the cancer cells or the tumor-associated stromal cells reported previously [14]. Because each tumor could be unique in certain aspects we chose to determine differentially expressed genes by HTself data analysis rather than treating them as biological replicates of each other. The CD26^+ ^cancer datasets obtained were representative of Gleason grades 3 and 4. Grade 3 tumors show a glandular morphology whereas grade 4 tumors are typically less glandular. Sorting for grade 5 is problematic as Gleason 5+5 tumors are rarely seen. According to many pathologists, cells of Gleason grade 3 are those that are first recognizable as cancerous. Compared to the normal counterpart luminal cells, the expression of a variety of genes was found to be different. This could be due to the altered expression of several transcription factors. Some of the genes identified are involved in cell division and response to apoptotic signaling. Genes of the Wnt pathway, which is important in epithelial-stromal cell interaction, appeared to be dysregulated to produce increased Wnt signaling in the cancer cells. Targeting this particular pathway may prove effective in prostate cancer.

The cancer cell types profiled in this study were the CD10^- ^luminal-like types, although one populated a Gleason 3+3 tumor and the other a Gleason 4+4 tumor. Their respective transcriptomes represent the signatures of two distinct prostate cancer cell types. As greater numbers of cancer cell types from different cases are analyzed, it will be interesting to determine if dissimilarities in gene expression increase with higher Gleason grades. This could be visualized using PCA. The number of possible prostate cancer cell types is currently unknown, and tumor behavior could be dependent on its composition of various cancer cell types. For example, the CD10^+ ^type is associated with lymph node metastasis and poor outcomes [8,45]. Isolation of this cell type is hampered by the fact that luminal cells are also CD10^+ ^and CD10 staining of biopsy is currently not a standard practice. Other cell types like CD44^+ ^[46] are even more rarely encountered.

The identification of candidate biomarkers for cancer detection and disease stratification in this study is of practical utility. Their identity would provide for a targeted approach to analyze for their presence in body fluids such as urine and blood. Genes that encode secreted proteins are particularly useful as most, if not all, clinical tests are based on immunodetection of these types of molecules. Since cancer cells are non-functional, it was not expected that many such proteins would be found. As can be inferred from the array signal intensity levels, expression of the major functional prostatic secretory proteins ACPP, AZGP1, KLK2, KLK3, and MSMB was down-regulated in the cancer cells compared to the luminal cells. Of the candidates, AGR2 appeared most promising. AGR2 is a relatively small protein and is produced apparently at a high level based on the array signals (>4000). AGR2 was also readily detectable in the tissue digestion media of cancer specimens by Western blot analysis. AGR2 expression may be decreased in higher Gleason tumors. Thus, AGR2 could potentially serve as a target biomarker for early disease detection. In contrast, the secreted CCL3/CCL4 could serve as a biomarker of tumors containing a G4 cancer cell type. Care must be taken to ensure that these potential biomarker proteins are not produced significantly by other cell types. Expression of these candidates will be examined in greater detail by tissue microarray analysis. A multimarker panel could conceivably be generated to incorporate all informative genes to diagnose prostate cancer.

## Conclusions

In summary, we have determined the transcriptomes of CD26^+ ^primary prostate cancer cells isolated from Gleason grade 3 and grade 4. Grade 4 is usually associated with poor clinical outcomes. Therefore, genes differentially expressed between these two grades have prognostic potential. The method we have described to isolate primary prostate cancer cells for transcriptome analysis should be applicable to other solid tumors.

## Competing interests

The authors declare that they have no competing interests.

## Authors' contributions

LEP and AYL designed research; LEP, LSP, ESL, CPS, PT, BM, and AYL performed research; LDT provided pathology analysis, assisted with study design and helped write the manuscript. LEH assisted with study design. LEP, RZNV, and AYL analyzed data; LEP, RZNV and AYL wrote the manuscript with contribution from the coauthors. All authors read and approved the final manuscript.

## Pre-publication history

The pre-publication history for this paper can be accessed here:

http://www.biomedcentral.com/1471-2407/9/452/prepub

## Supplementary Material

Additional file 1 **Differentially expressed genes in cancer**. Differentially expressed genes between G3 CD26^+ ^cancer cells and CD26^+ ^luminal cells were identified by HTself. Genes with ≥8-fold difference in expression are listed. Entries in green were previously reported in other public datasets. ESTs are highlighted in tan. Wnt pathway genes are highlighted in yellow. Click here for file

Additional file 2 **Self-self experiment with HTself cutoff for differential expression**. The HTself method was applied to self-self experiments (orange points) with a 99.9% credibility interval to generate the intensity-dependent curve (green). The *x*-axis represents the RMA-normalized intensity average (*X*_i_/2 + *X*_j_/2) and the *y*-axis represents the logarithmic ratio (*X*_i _- *X*_j_) using self-self data (cancer *i vs*. cancer *j*). Actual data points (non-self-self) are compared against the intensity-dependent cutoffs. See ref. 18 for details. Click here for file

Additional file 3 **Differentially expressed genes in G3 and G4**. Differentially expressed genes between G3 CD26^+ ^cancer cells and G4 CD26^+ ^cancer cells were identified by HTself. Genes with ≥8-fold difference in expression are listed. Click here for file
