# Supplementary figures and images for: Gene expression relationship between prostate cancer cells of Gleason 3, 4 and normal epithelial cells as revealed by cell type-specific transcriptomes
Source: BMC Cancer. 2009 Dec 18;9:452. doi: 10.1186/1471-2407-9-452 (PMC2809079; doi:10.1186/1471-2407-9-452)

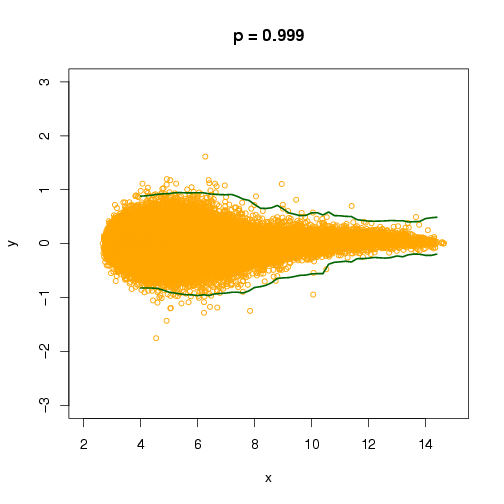

Supplement: Additional file 2 — Self-self experiment with HTself cutoff for differential expression. The HTself method was applied to self-self experiments (orange points) with a 99.9% credibility interval to generate the intensity-dependent curve (green). The x-axis represents the RMA-normalized intensity average (Xi/2 + Xj/2) and the y-axis represents the logarithmic ratio (Xi - Xj) using self-self data (cancer i vs. cancer j). Actual data points (non-self-self) are compared against the intensity-dependent cutoffs. See ref. 18 for details. [file 1471-2407-9-452-S2.PNG]
